# Consumer Anxiety and Assertive Advertisement Preference: The Mediating Effect of Cognitive Fluency

**DOI:** 10.3389/fpsyg.2022.880330

**Published:** 2022-05-30

**Authors:** Jun Wang

**Affiliations:** School of Economics and Management, Northeast Normal University, Changchun, China

**Keywords:** assertive advertising, consumer anxiety, effectiveness, attitude toward advertisement, preference, cognitive fluency

## Abstract

Companies often seek to persuade consumers to buy products or services through assertive advertising, but such advertising is often resisted by consumers. In order to identify ways to increase consumers' preference for assertive advertising, this study starts by considering consumers' anxiety and finds, through two between-group experiments, that the emotional state of consumers when viewing advertisements affects their attitudes toward assertive advertisements: anxious consumers have a more positive attitudes toward assertive advertisement, and cognitive fluency plays a mediating role in the relationship between consumer anxiety and consumer attitudes toward assertive advertisement. This study incorporates consumer anxiety into the study of assertive advertising, thus both enriching the theoretical research on assertive advertising and consumer anxiety and providing novel ideas for companies to enhance the effectiveness of their assertive advertising strategies.

## Introduction

Advertising is an important means by which companies seek to persuade consumers to buy products or services (Rana and Arora, 2021). In order to increase the persuasion effect, companies often use assertive advertising (Baek et al., 2015; Kim et al., 2017), with commanding rather than suggestive language that forces consumers to obey instructions (Miller et al., 2007). Notably, 72% of advertisements in the top 10 US print magazines contain assertive language such as “visit us,” “buy now,” and “call now” (Zemack-Rugar et al., 2017). Even well-known advertisements such as Nike's “Just Do It,” Sprite's “Obey Your Desire,” and Burger King's “As You Go” are assertive ads (Wang and Zhang, 2020). However, the existing literature on the effectiveness of assertive advertising is controversial. On the one hand, studies have found that consumers resist assertive advertising (Grandpre et al., 2003; Fitzsimons and Lehmann, 2004; Miller et al., 2007; Kronrod et al., 2012a), because such advertising creates a perception of coercion in consumers (Grandpre et al., 2003), creating pressure to conform, which in turn provokes resistance from audiences (Dillard and Shen, 2005; Pavey and Sparks, 2009), limiting the effectiveness of assertive advertising (Fitzsimons and Lehmann, 2004; Dillard and Shen, 2005; Quick and Considine, 2008). On the other hand, there are also studies showing that assertive advertising has obvious effects. In advertisements promoting, for example, hedonic products and environmental protection, the use of assertive advertising improves the persuasion effect (Kronrod et al., 2012a,b), and the persuasive effect will be affected by the consumer's level of self-confidence, perceived effort, cultural differences, non-compliance guilt, and other factors (Terlutter et al., 2010; Baek et al., 2015; Kim et al., 2017; Zemack-Rugar et al., 2017). It is evident that the success of assertive advertising depends on factors such as the content of the advertisement and the individual audience.

This paper takes emotion as another consumer individual factor, and explores whether assertive advertising has obvious persuasive effects on anxious consumers. Anxious consumers were identified as appropriate study subjects because of the large number of consumers who often experience transient anxiety in certain consumption situations, e.g., an inexperienced homebuyer who is concerned about losing the opportunity to buy a desirable home may feel anxious when making an offer, how they will invest and save; or how they will choose a medical plan (Gino et al., 2012). Anxiety often affects consumers' judgment and decision-making (Raghunathan and Pham, 1999); therefore, how to effectively target anxious consumers for advertising and marketing is particularly important. Furthermore, anxiety, as an emotion, is characterized by high uncertainty and low control (Smith and Ellsworth, 1985; Raghunathan and Pham, 1999), so individuals in anxious moods have a marked aversion to uncertainty (Maner et al., 2007). The message of assertive advertising is clear, easy to understand (Miller et al., 2007), and has obvious control (Reavey et al., 2018); therefore, we believe that the above characteristics of assertive advertising complement those of anxious consumers. Consistently, this match makes their preference for assertive advertising significantly higher than that of non-anxious consumers. This paper verifies this conjecture through experiments. In addition, we reveal the internal mechanism that causes consumers under anxiety to prefer assertive advertising, that is, the mediating effect of cognitive fluency. Cognitive fluency is an important factor in determining the effectiveness of advertising (Storme et al., 2015). We believe that the assertive language in advertisements helps anxious consumers under anxiety to perceive certainty. This matching makes anxious consumers more sensitive to assertive language. The cognitive processing of advertisements is more fluent, thus forming a positive advertisement attitude. Experiment 2 verifies our speculation.

Overall, this paper makes three theoretical contributions. First, this study incorporates consumer anxiety as an individual influencing factor into the research system, expanding the theoretical research on assertive advertising; second, the results enrich the theory of consumer anxiety and provides a clear understanding of which advertisements consumers prefer when under anxiety. Finally, this paper reveals the psychological mechanism of consumers' preference for assertive advertising under anxiety, that is, the mediating effect of cognitive fluency.

## Theoretical Review

### Advertisement Type

Advertisements can be classified into two types according to language intensity: assertive and non-assertive (Kronrod et al., 2012a,b; Zemack-Rugar et al., 2017; Wang and Zhang, 2020). Assertive advertisements use assertive language to persuade consumers, that is, by using verbs to directly issue orders to consumers, and adopting powerful adverbs such as “should” and “must” to clearly and directly tell consumers what to do, thus seeking to compel consumers to obey (Grandpre et al., 2003; Kronrod et al., 2012a; Baek et al., 2015). Assertive advertising is simple in form, clear in meaning, and suitable for persuading consumers (Kim et al., 2017). However, this type of advertising also creates pressure to obey, activates consumer resistance (Clee and Wicklund, 1980; Dillard and Shen, 2005; Lavoie et al., 2017; Wang and Yang, 2020), and even motivates the non-compliance guilt of disobedience, undermining the consumer-brand relationship (Zemack-Rugar et al., 2017).

For an assertive advertisement to have a positive effect, certain conditions must be met in actual application. From an advertising perspective, assertive advertising is more effective if the advertisement is anthropomorphic (Reavey et al., 2018); assertive advertising improves persuasion if the product advertised is a hedonic product (Kronrod et al., 2012b); if the content of the advertisement involves environmental protection themes: the use of assertive advertisements for urgent environmental protection appeals can arouse positive consumer perceptions (Kronrod et al., 2012a), and consumer perceived effort is key to the persuasiveness of assertive advertisements on environmental protection themes (Baek et al., 2015). From the perspective of cultural background, in countries with low self-confidence (such as Argentina), assertive advertising stimulates consumer self-confidence and produces better communication effects (Terlutter et al., 2010), whereas American consumers, for example, are likely to be more confident and more resistant to assertive advertisements than Korean consumers (Kim et al., 2017). In addition, the consumer perception of fit between assertive advertising and products drives consumer purchase intent (Wang and Zhang, 2020). To sum up, current research mainly focuses on advertising, cultural background, and individual consumer factors, while little is known about the role of consumer anxiety in assertive advertising.

### Consumer Anxiety

Anxiety is a subjective emotion that makes individuals consciously perceive fear and tension (Spielberger, 1966). This emotion signals the presence of a potential threat, triggering pessimistic assessments of future events and negative psychological responses (Butler and Mathews, 1983; Raghunathan and Pham, 1999; Shepperd et al., 2005). Previous studies have classified types of anxiety into trait anxiety and situation anxiety (also known as state anxiety) (Endler et al., 1991; Endler, 1997; Stöber, 1997), A-trait refers to a more stable predisposition or proneness to state anxiety, while A-state is conceptualized as a momentary or emotional state reaction accompanied by physiological arousal (Kantor et al., 2001), the difference between the two being the individual's own tendency to behave in a particular situation or an actual reaction in a particular situation (Endler and Shedletsky, 1973).

In consumer-behavior research, the form of consumer anxiety that many studies focus on is situational anxiety, which is a short-lived emotional state triggered by threatening situations, specifically manifested in the form of emotions such as fear, depression, stress, worry, and tension (Spielberger, 1966; Brooks and Schweitzer, 2011). The origin of consumer anxiety may be directly caused by an event or triggered by previous stimuli unrelated to the current decision (Raghunathan and Pham, 1999; Lerner and Keltner, 2001). In this paper, we focus on state anxiety, a transient emotion that anyone can experience. Over the last few decades, people have become more anxious, worrying about safety, social acceptance, and job security more than in the past, which has labeled the twentieth century “the age of anxiety.” (Twenge, 2000). In daily decision-making situations, many factors can make consumers anxious, such as technology anxiety (Meuter et al., 2003), stereotype threat anxiety (Lee et al., 2011), travel anxiety (Reisinger and Mavondo, 2005), death anxiety (Rahimah et al., 2018), math anxiety (Suri et al., 2013), and status anxiety (Chiou and Pan, 2008) employee anxiety (Xue et al., 2021). These even include some specific consumption situations, for example, an inexperienced homebuyer who is concerned about losing the opportunity to buy a desirable home may feel anxious when making an offer or how to invest savings; furthermore, people may feel anxious about how to choose a course of medical treatment (Gino et al., 2012). Anxiety is characterized by high uncertainty and low control (Smith and Ellsworth, 1985; Raghunathan and Pham, 1999). Therefore, rather than trait anxiety, we are more concerned about the influence of consumers on decision-making under state anxiety, and anxious consumers tend to make decisions that promote a sense of security and self-control (Raghunathan et al., 2006) such as compulsive buying (Darrat et al., 2016). Studies have shown that consumer emotional factors play a role in advertising persuasion (Lau-Gesk and Meyers-Levy, 2009), motivate and persuade consumers, and often guide people's attitudes and behaviors (Andrade and Cohen, 2007). Existing research does not shed light on the form of advertising that anxious consumers prefer. The focus of this paper, thus, is how—when facing the powerful persuasion effects of assertive advertisements—consumers under anxiety make decisions, and what attitudes they have toward such advertisements.

### Cognitive Fluency

Cognitive fluency, which is broadly defined as the ease by which a stimulus can be perceived, processed, or retrieved (Hoorens and Bruckmüller, 2015), involves subjective fluency, which varies according to the stimuli, external environment, and individual perception and conceptual fluency (Alter and Oppenheimer, 2009). Cognitive fluency is embodied in the fluency of concepts, language, space, perception, and decision-making (Oppenheimer, 2008). Among these, perceptual fluency is caused by lower-order cognition and is related to the presentation of stimuli, while conceptual fluency involves higher-order cognitive processes related to recognition of the relationship between the form and the content of the stimulus, the significance of the context, or the stimulus classification (Cabeza and Ohta, 1993). Greater fluency generally provokes favorable responses, giving consumers the perception of ease of processing, low effort, and high efficiency (Reber et al., 2004), which increases their subjective preference for stimuli (Winkielman et al., 2003), positive evaluation (Shen et al., 2008), and trust (Schwarz et al., 2021). Cognitive fluency is applied in the field of advertising. Important cognitive processes related to consumer-related subjective experience help improve image fluency and facilitate the understanding of narrative advertising (Chang, 2013). Cognitive fluency improves consumers' fluency in processing advertising information. The face of a disfluent celebrity in an advertisement affects brand memory (Liu and Liu, 2020). The more smoothly the advertisements are processed, the easier it is to stimulate consumption. attention, processing motivation, and processing depth, among others, to improve advertising attitudes and increase purchase intention (Storme et al., 2015).

## Research Hypothesis

### Consumer Anxiety, Assertive Advertising and Advertising Attitudes

Consumers generally perceive assertive advertising to be sales-oriented, using strong language to “hard sell.” This approach not only makes consumers perceive rudeness (Roberts and Kreuz, 1994; Dillard et al., 1997) but also a form of public persuasion (Zemack-Rugar et al., 2017). Consumers have accumulated knowledge in their long-term interactions with various marketing stimuli, making them adept at identifying marketing persuasion techniques that they perceive as reflecting the advertiser's manipulative intent (Campbell, 1995). The persuasive knowledge model suggests that consumers have negative perceptions of advertising when they realize that they are being manipulated (Campbell and Kirmani, 2000), and that consumers' consciously formed advertising perceptions ultimately shape advertising attitudes (Shimp, 1981). Attitude toward advertising refers to the tendency of consumers to respond favorably or unfavorably to specific advertising stimuli (MacKenzie and Lutz, 1989), and, based on this, we infer that consumers will question the intentions of advertisers due to assertive advertising. This leads to psychological resistance, which leads to a negative attitude toward assertive advertising.

Advertising language often expresses multi-layered and ambiguous meanings, and consumers must use inductive reasoning to process this information (Reavey et al., 2018). However, anxious consumers are confronted with a large number of advertisements every day, and cannot process this information effectively (Marquez, 1977) because of their cognitive exhaustion which leads to a decline in their ability to interpret and comprehend information (Sengupta and Johar, 2001). As a result, they prefer safe and high-control options (Taylor, 1974; Raghunathan et al., 2006), and avoid risk and uncertainty even when the decision-making task in advertising is not associated with anxiety-inducing stimuli (Brooks and Schweitzer, 2011). In the interaction of consumers with advertisements in the face of such emotion, they are more inclined to collect information from advertisements and use it as cues to make decisions (Gino et al., 2012). Compared with non-assertive advertising, the persuasive message of assertive advertising is considered clear and easy to understand (Miller et al., 2007), which reduces the low sense of control and uncertainty caused by the advertising message. Consumers are guided by direct and clear actions, thus creating a stronger preference for assertive advertising among anxious consumers than non-anxious ones. Based on this, we propose the following hypothesis:

H1: Anxious consumers have more positive attitudes toward assertive advertisement than non-anxious consumers.

### The Mediating Role of Cognitive Fluency

Consumers can clarify their purchase intentions through advertising information and preferences. Assertive advertisements often use imperative adverbs such as “should” and “must” (Baek et al., 2015), making consumers feel compelled to follow certain instructions (Okazaki et al., 2010) that are difficult to reject (Kronrod et al., 2012a,b). Therefore, the consumer's behavioral tendency and the explicit instruction of the assertive advertisement form a double message, which affects the cognitive fluency. Moreover, this approach makes them feel that their freedom and autonomy are threatened (Dillard and Shen, 2005), and consumers will refuse to comply with a strong motivation to protect personal freedom (Clee and Wicklund, 1980; Dillard and Shen, 2005; Pavey and Sparks, 2009), and even induce resistive behaviors (Clee and Wicklund, 1980; Dillard and Shen, 2005; Lavoie et al., 2017; Wang and Yang, 2020). In contrast, when facing important decisions, anxious consumers feel uncertain about their ability to make the right decisions, lack confidence in their own judgment (Gino et al., 2012), and tend to think that they are in a non-dominant position and lacking control (Maddux et al., 1988). When consumers form judgments and make decisions, they are more inclined to respond to information that is easy to process (Shah and Oppenheimer, 2007), and when the content of the information is concise, it is easier to stimulate consumers' cognitive fluency (Novemsky et al., 2007). Assertive advertising gives consumers clear instruction in action decision-making, and such advertising information is more convincing (O'Keefe, 1997), which makes the direction of action clear, thereby increasing the cognitive fluency of advertising. The more smoothly the advertising information is recognized, the easier it is to capture consumers' attention, processing motivation, and processing depth, thereby enhancing consumers' attitudes toward advertising (Storme et al., 2015). Based on this, we propose the following hypothesis:

H2: Cognitive fluency plays a mediating role in the effects of consumer anxiety and advertising type on consumer advertising attitudes.
H2a: Anxious consumers have higher cognitive fluency with assertive advertisement and thus have more positive attitudes toward such advertisement.
H2b: Non-anxious consumers have higher cognitive fluency with non-assertive advertisement and thus have more positive attitudes toward such advertisement.

**Figure 1:**
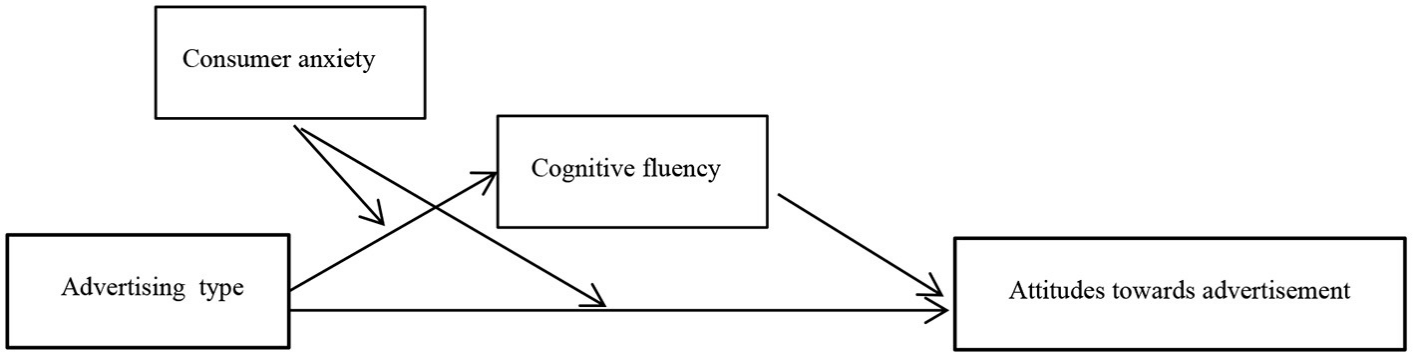
The conceptual model of this study.

The conceptual model of this study is shown in Figure 1.

## Experiment 1

### Pre-experiment

The purpose of the pre-experiment is to select suitable material for assertive advertising and anxiety emotion manipulation in the formal experiment. In terms of advertising, according to the experimental method of Kronrod et al. (2012b), this paper selects the virtual brand BMY earphones that offer both practical and hedonic value as advertised products.The advertising language of one of the assertive advertisements was “BMY earphones, you must buy”and that of the other, i.e. the non-assertive advertisement, was “BMY earphones are worth trying.” Sixty subjects were invited to rate the assertiveness of the two advertising phrases using the manipulative question “I feel that someone is ordering me to take action in this advertisement” on a 7-point Likert scale (1 = strongly disagree, 7 = strongly agree). The assertiveness of the assertive advertisement (M assertive = 4.83, SD assertive = 0.75) was statistically significantly higher than that of the non-assertive advertisement (M non-assertive = 1.00, SD non-assertive = 0.00, *t*(29) = 28.12, *p* < 0.01); *t*-tests were performed under heteroscedasticity conditions; as a result, df are different to those expected for a homovariance *t*-test. Therefore, the two advertisements were identified as stimulant materials for the advertising experiments of this study.

In accordance with Brooks and Schweitzer (2011) and Gino et al. (2012), to manipulate the anxiety of consumers, a clip from the movie “Vertical Limit^1^” “(between 0 min 29 s and 6 min 25 s) is used induce to anxiety, while in the non-anxiety group, we used a clip from the BBC documentary” The Great Barrier Reef^2^” (between 0 min 0 s and 5 min 0 s) to induce neutral emotions. Similarly, 60 subjects were invited to score the anxiety-evoking degree of the two videos. The manipulative questions used were based on the personal emotional scale compiled by Raghunathan and Pham (1999), and the item “I felt anxious” was selected as the subject. The anxiety manipulative test questions were measured using a 7-point Likert scale (1 = strongly disagree, 7 = strongly agree). The final movie “Vertical Limit” segment induced statistically significantly higher anxiety (M anxiety = 6.30, SD anxiety = 0.70) than the BBC documentary “Great Barrier Reef” segment (M non-anxiety = 1.23, SD non-anxiety = 0.43, *t*(34) = 48.10, *p* < 0.01); *t*-tests were performed under heteroscedasticity conditions; as a result, df are different to those expected for a homovariance *t*-test, and the two movies were selected as the emotional-manipulation experimental material of this study.

### Formal Experiment

The purpose of Experiment 1 is to verify the difference in attitude between anxious consumers and non-anxious consumers toward assertive advertising, namely, to test hypothesis 1.

#### Procedure and Measurement

This study adopted a 2 (advertising type: assertive advertising vs. non-assertive advertising) × 2 (consumer anxiety: anxiety vs. non-anxiety) between-group experimental design. A total of 200 college students were recruited from a university in Northeast China to participate in this experiment. The final valid sample was 188 (average age was 19.6 years old, and 82 were female, accounting for 43.6%). All subjects were randomly assigned to one of four conditions corresponding to a between-subjects design. First, the anxiety group was asked to watch the clip from the movie “Vertical Limit” (0 min 29 s to 6 min 25 s) to induce anxiety, while the non-anxious group watched the BBC documentary “The Great Barrier Reef” (0 min 0 s to 5 min 0 s) to induce a neutral emotion, and then answer the question “I feel anxious” on the anxiety-manipulation test. Next, subjects in both the assertive and the non-assertive advertisement group were asked to view the corresponding advertising materials and to complete the manipulative test question for the assertive ads: “From this ad, I feel that someone is ordering me to take action,” and fill in the measurement items for advertising attitude. To measure advertising attitude, in accordance with the advertising attitude scale compiled by MacKenzie and Lutz (1989) and Lee et al. (2005), combined with the research context, three items were determined: “I feel this advertisement is good,” “This advertisement attracts me,” and “I like this advertisement.” (Cronbach's α = 0.96). All measurements are on a 7-point Likert scale (1 = strongly disagree, 7 = strongly agree). Finally, subjects filled in basic information questions to complete the experiment. The subjects were rewarded with a small gift after completing all the items.

#### Results and Discussion

##### Manipulation Checks

The independent sample *t*-test on consumer anxious types and advertising types showed that the anxiety score of the anxiety group (M anxiety = 5.37, SD anxiety = 1.43) was statistically significantly higher than that of the non-anxiety group (M non-anxiety = 2.60, SD non-anxiety = 1.43, *t*(186) = 13.19, *p* < 0.01); the scores of assertive advertisement groups (M assertive = 5.55, SD assertive = 1.56) were statistically significantly higher than those of non-assertive advertisement groups (M non-assertive = 1.65, SD non-assertive = 0.85, *t*(150) = 21.52, *p* < 0.01 e advertisement groups (M non-assertive = 1.65, SD non-assertive = 0.85, *t*(150) = 21.52, *p* < 0.01); *t*-tests were performed under heteroscedasticity conditions; as a result, df are different to those expected for a homovariance *t*-test, indicating that the manipulation was successful.

##### Main Effect Analysis

**Figure 2:**
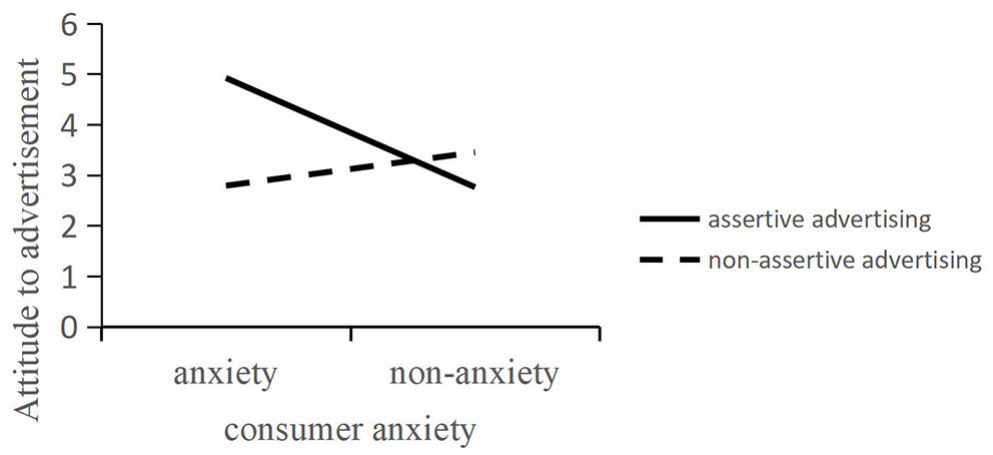
The interaction between advertising types and consumer anxiety in Experiment 1.

The main effect analysis shows that the main effect of assertive advertising on advertising attitude is significant [*F*(1, 184) = 12.84, *p* < 0.01], and consumer anxiety has a significant impact on advertising as evident from analyzing the variance of consumer anxiety, advertising type, and advertising attitude. The main effect was significant [*F*(1, 184) = 14.05, *p* < 0.01], and the interaction effect between assertive advertising and consumer anxiety was significant [*F*(1, 184) = 49.92, *p* < 0.01]. So, hypothesis 1 was supported. A simple effect analysis was carried out by writing statements in SPSS to verify how the interaction of advertising types and consumer sentiment affects advertising attitudes. The results show that consumers under anxiety have a more positive attitude toward advertising [M assertive = 4.92; SD assertive = 0.22; M non-assertive = 2.79; SD non-assertive = 0.21; *F*(1, 184) = 49.90, *p* < 0.01]; non-anxious consumers have more positive attitudes toward non-assertive advertising [M non-assertive = 3.45; SD non-assertive = 0.19; M assertive = 2.76; SD assertive = 0.18; *F*(1, 184) = 7.01, *p* < 0.01,power analysis =0.926; effect size=0.264], as shown in Figure 2; therefore, hypothesis 1 was confirmed.

## Experiment 2

The purpose of Experiment 2 was to verify the mediating effect of cognitive fluency on consumer anxiety, advertising type, and advertising attitude, namely, to test hypothesis 2,2a, and 2b.

### Procedure and Measurement

A total of 200 college students were recruited to participate in this experiment from a university in Northeast China, and 187 subjects finally completed the experiment (the average age was 19.17 years, and 92 were women, accounting for 49%). The procedures for Experiment 2 and Experiment 1 were basically the same. All subjects were randomly assigned to one of four conditions corresponding to a between-subjects design. The subjects were required to complete the test questions about the manipulativeness of assertive advertisements and then complete the cognitive fluency measurement scale. The measurement of cognitive fluency was based on Chae and Hoegg (2013) and Hoorens and Bruckmüller (2015), and comprised the following three items, “This advertisement is very simple,” “This advertisement is easy to understand,” and “I understand this advertisement very clearly.” Finally, participants answered questions on advertisement attitude and provided basic information such as their age and gender. The subjects were rewarded with a small gift after completing all the items.

### Results and Discussion

#### Manipulation Checks

The results of independent samples *t*-test on consumer types and advertising types showed that the emotional score of the anxiety group (M anxiety = 5.91, SD anxiety = 1.09) was statistically significantly higher than that of the non-anxious group [M non-anxious = 1.51, SD non-anxious = 0.10, *t*(185) = 28.81, *p* < 0.01]; the assertive score of the advertisements in the assertive advertisements group (M assertive = 5.35, SD assertive = 1.90) was statistically significantly higher than that of the non-assertive advertisements group [M non-assertive = 2.48, SD non-assertive = 1.60, *t*(185) = 10.91, *p* < 0.01], indicating that the experimental manipulation was successful.

#### Main Effect Analysis

**Figure 3:**
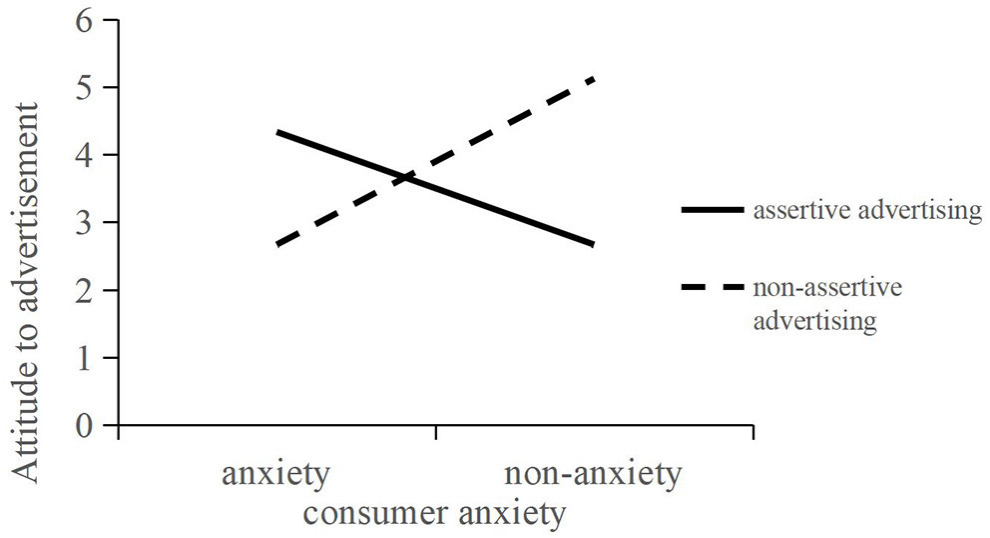
The interaction between advertising types and consumer anxiety in Experiment 2.

Analysis of variance on consumer anxiety, advertising type, and advertising attitude showed that the main effects of assertive advertising [*F*(1, 183) = 4.01, *p* = 0.047] and consumer anxiety [*F*(1, 183) = 4.03, *p* = 0.046] on advertising attitude were both statistically significant; furthermore, the 2 (anxiety vs. non-anxiety) × 2 (assertive advertising vs. non-assertive advertising) analysis of variance (ANOVA) revealed a significant main effect of advertising attitude [*F*(1, 183) = 5.83, *p* < 0.01], re-confirming Hypothesis 1. In order to further understand how advertising types and consumer anxiety affect advertising attitudes, this study used SPSS to conduct a simple effect analysis. The results showed that consumers in an anxious emotion had a more positive attitude toward assertive advertisement (vs. non-assertive advertisement) [M assertive = 4.33; SD assertive = 0.19; M non-assertive = 2.66; SD non-assertive =0.18;*F*(1, 183) = 37.78, *p* < 0.01]; non-anxious consumer advertising attitudes toward non-assertive advertisement (vs. assertive advertisement) more positive [M non-assertive = 5.12; SD non-assertive =0.20;M assertive = 2.66; SD assertive =0.19;*F*(1, 183) = 79.56, *p* < 0.01, power analysis =0.925; effect size=0.265], as shown in Figure 3.

#### Mediating Effect Analysis

In accordance with the mediation effect analysis procedure proposed by Zhao et al. (2010) and Shi et al. (2011), we use a step-by-step process for testing mediation. Furthermore, referring to the bootstrap method proposed by Preacher et al. (2007) and Hayes (2013), the PROCESS program in SPSS was used to test the mediation effect. This paper uses the process model 8 in SPSS to test the mediating effect of cognitive fluency (Cronbach's α = 0.84) effect, and the sample size was set to 5,000. The results showed that, with the advertising attitude as the dependent variable, the mediating effect of cognitive fluency was statistically significant (LLCI = 1.43, ULCI = 2.55, excluding 0) under the 95% confidence interval, and the effect coefficient was 1.95. Specifically, the mediating effect of cognitive fluency in the influence of assertive advertising and consumer anxiety on advertising attitudes is statistically significant (LLCI = −1.38, ULCI = −0.64, excluding 0). The mediating effect of cognitive fluency in the influence of non-assertive advertising and non-anxious emotion on advertising attitudes was statistically significant (LLCI = 0.66, ULCI = 1.32, excluding 0), thus verifying hypothesis 2, 2a and 2b.

## Discussion and Conclusion

In this paper, two experiments were conducted to verify the influence of the interaction of consumer anxiety (anxiety vs. non-anxiety) and advertising type (assertive advertising vs. non-assertive advertising) on advertising attitudes and the mediating effect of cognitive fluency. Specifically, the results of Experiment 1 show that when consumers are anxious, using assertive advertising can improve consumer advertising attitudes more than non-assertive advertising; and when consumers are in a non-anxious state, using non-assertive advertising is more effective than non-assertive advertising. Assertive advertising can improve consumer attitude toward advertising.

The results of Experiment 2 further showed that cognitive fluency played a mediating role in the interaction effect of consumer anxiety (anxiety vs. non-anxiety) and advertising type (assertive advertising vs. non-assertive advertising) on advertising attitudes. Anxious consumers have higher cognitive fluency for assertive advertisements, and thus have a more positive attitude toward assertive advertisements; non-anxious consumers have higher cognitive fluency toward non-assertive advertisements, so they are more responsive to non-assertive advertisements. The attitude of the advertising is more positive.

### Implications

The theoretical contributions of this study are mainly reflected in three aspects. First, the conclusions of this paper enrich the research on assertive advertising. In regard to the benefits and disadvantages of assertive advertising, previous researchers have been divided in their opinions; many have discussed the effectiveness of assertive advertising in various specific situations, but few studies have examined it from the perspective of consumer anxiety. This paper takes a novel perspective, namely the anxiety of consumers, and discusses the attitudes of consumers under anxiety to assertive advertising, thereby expanding the research boundary of the effectiveness of assertive advertising. Second, this paper enriches the research on consumer anxiety theory. Previous research has mainly focused on the drivers of consumer anxiety and its impact on decision-making, but little work has been done on the impact of advertising attitudes. This paper takes anxious consumers as the research object, and discusses their response to assertive advertising, which provides a theoretical basis for appropriate advertising types for this group and also deepens the theoretical system of consumer anxiety. Finally, this paper reveals the psychological mechanism of consumer preference for assertive advertising under anxiety, that is, the mediating effect of cognitive fluency, thus offering an in-depth exploration of the psychological mechanism underlying the influence of assertive advertising on consumer advertising attitudes.

This paper provides suggestions for corporate marketing strategies. The findings provide ideas for marketers to design advertisements in different language styles. Consumers who are anxious prefer assertive advertisements; therefore, when designing advertisements, attention should be paid to gaining the favor of these consumers through assertive language and cues. In the actual marketing environment, we acknowledge that it is currently difficult to directly identify an anxious customer; despite this, it can be applied in situations that are likely to elicit anxiety (such as buying a house or making insurance investment), and our conclusions are valuable when purchasing such products or services. Advertisements should use more slogans to emphasize clear and short action instructions, which can more effectively reduce hesitation of anxious consumers in purchasing decisions and alleviate the uncertainty caused by the lack of instructions in advertising information. Furthermore, the results provide insights for the enterprise market segmentation strategy. The market research department of an enterprise can measure the anxiety of consumers through questionnaires, and then formulate different types of advertisements for different market segments according to the results. In international marketing, different market categories can be divided explicitly according to the anxiety level of consumers in the target market in a cross-cultural context. In certain market situations such as purchasing complex products (such as automobiles, high-tech products) (Lee et al., 2011) or less familiar products (such as insurance investments, real estate) (Gino et al., 2012), it is more likely to induce consumer anxiety. In these segmented market where consumers are prone to anxiety, assertive advertising should be the main method, and attention should also be paid to activating high uncertainty or low sense of control (flash sale) among consumers during the publicity process so as to achieve better persuasive effects.

### Limitations

This paper is the first to explore consumer preference for arbitrary advertising from the perspective of consumer anxiety, so there are still some limitations and problems to be addressed. First, this paper only studies attitudes toward assertive advertisement under the boundary condition of consumer anxiety; there may be other boundary conditions, such as product type. The subjects in the experiment in this paper are college students; accordingly, the experimental products selected are headphones, which are both hedonic and practical. However, in fact, when hedonic or practical products are more described as hedonic functions, assertive advertising will lead to greater compliance of consumers (Kronrod et al., 2012b), Therefore, for anxious consumers, whether there is still a difference in the persuasive effect of utility and hedonic products in assertive advertising remains to be further explored. Second, our experimental sample has limitations. In order to ensure more rigorous control of state anxiety, laboratory samples were used in both Experiment 1 and Experiment 2, and random sampling was not carried out in different age groups. Therefore, follow-up research can use a wider sample for further verification. We also suggest that future studies aim to perform verification and generalization of the present results. Finally, in addition to the print advertisements discussed in this article, assertive language is increasingly used in online live broadcasts, and the language of network anchors has begun to show an assertive trend, thus forming a generalization of the stimulation of assertive language whether anxious consumers also have preferences in this regard remains to be studied.

## Data Availability Statement

The original contributions presented in the study are included in the article/Supplementary Material, further inquiries can be directed to the corresponding author.

## Author Contributions

JW finished all steps of the study and performed the statistical analysis and revised the manuscript and contributed to the article and approved the submitted version.

## Conflict of Interest

The author declares that the research was conducted in the absence of any commercial or financial relationships that could be construed as a potential conflict of interest.

## Publisher's Note

All claims expressed in this article are solely those of the authors and do not necessarily represent those of their affiliated organizations, or those of the publisher, the editors and the reviewers. Any product that may be evaluated in this article, or claim that may be made by its manufacturer, is not guaranteed or endorsed by the publisher.

## Footnotes

^1^The video was obtained from a paid website in China, whose url is https://www.youku.com/show_page/id_zcc00e198962411de83b1.html.

^2^The video was obtained from a paid website in China, whose url is https://v.youku.com/v_show/id_XMTY2Mjc1Njc2MA==.html?spm=a2h0c.8166622.PhoneSokuProgram_1.dtitle&s=51a675b038e211e6bdbb.
